# *catena*-Poly[[diphenyl­propyl­tin(IV)]-μ-chloroacetato-κ^2^ *O*:*O*′]

**DOI:** 10.1107/S160053680802028X

**Published:** 2008-07-09

**Authors:** Mostafa M. Amini, Taraneh Hajiashrafi, Hamid Reza Khavasi, Ali Nematin Kharat

**Affiliations:** a Department of Chemistry, Shahid Beheshti University, Evin, Tehran 1983963113, Iran; b School of Chemistry, University College of Science, University of Tehran, Tehran, Iran

## Abstract

The title compound, [Sn(C_3_H_7_)(C_6_H_5_)_2_(C_2_H_2_ClO_2_)]_*n*_, comprises polymeric carboxyl­ate-bridged spiral chains with two monomer formula units in the asymmetric unit. Both Sn centres exhibit similar distorted trigonal–bipyramidal [C_3_SnO_2_] coordination, with the O atoms of the carboyxlate ligands in *trans* positions.

## Related literature

For the structures of similar organotin compounds, see: Amini *et al.* (2002 ▶, 2006 ▶); Ng *et al.* (1988 ▶); Teo *et al.* (2004 ▶). The synthesis of the precursor diphenyl-*n*-propyl­tin(IV) iodide follows the procedure given by Snegur & Manulkin (1964 ▶).
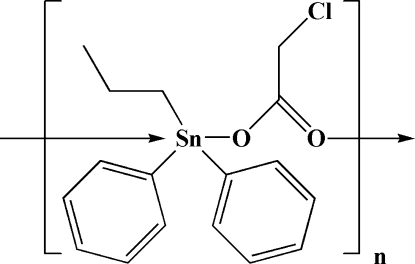


## Experimental

### 

#### Crystal data

[Sn(C_3_H_7_)(C_6_H_5_)_2_(C_2_H_2_ClO_2_)]
*M* *_r_* = 409.48
Orthorhombic, 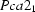

*a* = 19.330 (4) Å
*b* = 11.440 (2) Å
*c* = 16.390 (3) Å
*V* = 3624.4 (13) Å^3^
*Z* = 8
Mo *K*α radiation
μ = 1.56 mm^−1^
*T* = 298 (2) K
0.49 × 0.21 × 0.19 mm

#### Data collection

Stoe IPDSII diffractometer
Absorption correction: numerical [*X-RED* and *X-SHAPE* (Stoe & Cie, 2005 ▶)] *T*_min_ = 0.679, *T*_max_ = 0.826
13588 measured reflections
8672 independent reflections
7285 reflections with *I* > 2σ(*I*)
*R*_int_ = 0.034

#### Refinement

*R*[*F* ^2^ > 2σ(*F* ^2^)] = 0.045
*wR*(*F* ^2^) = 0.123
*S* = 1.11
8672 reflections
380 parameters
1 restraint
H-atom parameters constrained
Δρ_max_ = 0.79 e Å^−3^
Δρ_min_ = −0.55 e Å^−3^
Absolute structure: Flack (1983 ▶), with 3643 Friedel pairs
Flack parameter: −0.04 (4)

### 

Data collection: *X-AREA* (Stoe & Cie, 2005 ▶); cell refinement: *X-AREA*; data reduction: *X-AREA* (Stoe & Cie, 2005 ▶); program(s) used to solve structure: *SHELXS97* (Sheldrick, 2008 ▶); program(s) used to refine structure: *SHELXL97* (Sheldrick, 2008 ▶); molecular graphics: *ORTEP-3 for Windows* (Farrugia, 1997 ▶); software used to prepare material for publication: *WinGX* (Farrugia, 1999 ▶).

## Supplementary Material

Crystal structure: contains datablocks global, I. DOI: 10.1107/S160053680802028X/wm2181sup1.cif

Structure factors: contains datablocks I. DOI: 10.1107/S160053680802028X/wm2181Isup2.hkl

Additional supplementary materials:  crystallographic information; 3D view; checkCIF report

## Figures and Tables

**Table 1:** Selected bond lengths (Å)

Sn1—C3	2.105 (9)
Sn1—C12	2.125 (7)
Sn1—C6	2.137 (7)
Sn1—O1	2.178 (4)
Sn1—O4^i^	2.478 (5)
Sn2—C29	2.129 (7)
Sn2—C23	2.130 (6)
Sn2—C20	2.130 (8)
Sn2—O3	2.190 (5)
Sn2—O2	2.469 (5)

Symmetry code: (i) 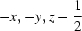.

**Fig. 1.**
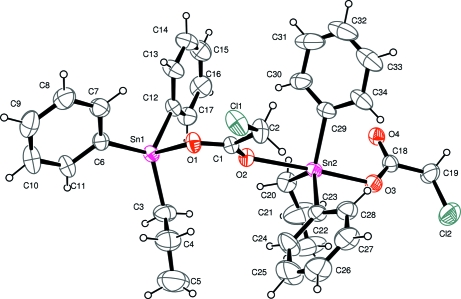
The asymmetric unit of the title compound as a portion of the polymeric chain, showing the atom labelling scheme and displacement ellipsoids drawn at the 30% probability level.

## supplementary crystallographic information

### Comment

This study continues our structural work on Sn compounds of carboxylic acid derivatives with mixed organic groups. Since the synthesis of these compounds (mixed organic groups on tin) is not always straightforward, only a few structures have been reported. For mixed aryl/alkyl carboxylates the structures of diphenylmethyltin (Amini *et al.*, 2006), diphenylmethyltin (Amini *et al.*, 2002) and diphenylcyclopentyltin (Teo *et al.*, 2004) are known. For all these structures, the Sn centres are carboxylate-bridged (Ng *et al.*, 1988). This structural motif is also realised in the title compound which consists of two independent molecules in the asymmetric unit (Fig. 1). The two molecules are linked via carboxylate O atoms to form polymeric spiral chains parallel to [001]. The distorted trigonal bipyrimidal [C_3_SnO_2_] coordination of the two Sn centers in the independent molecules is very similar, with one axial Sn—O bond being considerably longer (≈ 2.47 Å) than the other axial Sn—O bond (≈ 2.18 Å; Table 1).

### Experimental

Triphenyl-n-propyltin was prepared by using a conventional Grignard reaction based on triphenyltin chloride. The reagent was then treated with elemental iodine in order to cleave one of the three aromatic groups to yield diphenyl-n-propyltin(IV) iodide (Snegur & Manulkin, 1964). The latter (0.20 g, 0.45 mmol) was combined with silver monochloroacetate (0.09 g, 0.45 mmol) in methanol (10 ml), and the resulting AgI precipitate was removed by filtration. The solid obtained from concentration of the filtrate was purified by recrystallisation from a methanol–hexane (1:1 *v*/*v*) mixture to yield block-shaped, colorless crystals with an average size of about 0.3 mm (melting point 385 K).

### Refinement

The H atoms were placed in calculated positions (C—H = 0.93–0.97 Å) and included in the refinement in the riding model approximation, with their displacement parameters set at 1.2 or 1.5 (propyl) times *U*_eq_(C) of their parent atom.

### Crystal data

**Table:**

[Sn(C_3_H_7_)(C_6_H_5_)_2_(C_2_H_2_ClO_2_)]	*F*_000_ = 1632
*M**_r_* = 409.48	*D*_x_ = 1.501 Mg m^−^^3^
Orthorhombic, *P**c**a*2_1_	Mo *K*α radiation λ = 0.71073 Å
Hall symbol: P 2c -2ac	Cell parameters from 7531 reflections
*a* = 19.330 (4) Å	θ = 2.1–29.3º
*b* = 11.440 (2) Å	µ = 1.56 mm^−^^1^
*c* = 16.390 (3) Å	*T* = 298 (2) K
*V* = 3624.4 (13) Å^3^	Block, colourless
*Z* = 8	0.49 × 0.21 × 0.19 mm

### Data collection

**Table:**

Stoe IPDSII diffractometer	*R*_int_ = 0.034
ω scans	θ_max_ = 29.3º
Absorption correction: numerical[X-RED and X-SHAPE (Stoe & Cie, 2005)]	θ_min_ = 2.1º
*T*_min_ = 0.679, *T*_max_ = 0.826	*h* = −26→19
13588 measured reflections	*k* = −11→15
8672 independent reflections	*l* = −22→20
7285 reflections with *I* > 2σ(*I*)	

### Refinement

**Table:**

Refinement on *F*^2^	H-atom parameters constrained
Least-squares matrix: full	*w* = 1/[σ^2^(*F*_o_^2^) + (0.0589*P*)^2^ + 2.5701*P*] where *P* = (*F*_o_^2^ + 2*F*_c_^2^)/3
*R*[*F*^2^ > 2σ(*F*^2^)] = 0.045	(Δ/σ)_max_ = 0.026
*wR*(*F*^2^) = 0.123	Δρ_max_ = 0.79 e Å^−^^3^
*S* = 1.12	Δρ_min_ = −0.55 e Å^−^^3^
8672 reflections	Extinction correction: none
380 parameters	Absolute structure: Flack (1983), with 3643 Friedel pairs
1 restraint	Flack parameter: −0.04 (4)

### Special details

**Table:**

Geometry. All e.s.d.'s (except the e.s.d. in the dihedral angle between two l.s. planes) are estimated using the full covariance matrix. The cell e.s.d.'s are taken into account individually in the estimation of e.s.d.'s in distances, angles and torsion angles; correlations between e.s.d.'s in cell parameters are only used when they are defined by crystal symmetry. An approximate (isotropic) treatment of cell e.s.d.'s is used for estimating e.s.d.'s involving l.s. planes.

### Fractional atomic coordinates and isotropic or equivalent isotropic displacement parameters (Å^2^)

**Table:**

	*x*	*y*	*z*	*U*_iso_*/*U*_eq_	
Sn1	0.116980 (19)	−0.14033 (4)	−0.14385 (3)	0.06080 (11)	
Sn2	0.069248 (18)	0.19052 (3)	0.10089 (3)	0.05654 (10)	
Cl1	0.31159 (9)	−0.0258 (3)	0.04475 (13)	0.0973 (7)	
Cl2	0.0078 (3)	0.5469 (3)	0.2698 (3)	0.181 (2)	
O1	0.1911 (2)	−0.0768 (5)	−0.0541 (3)	0.0688 (11)	
O2	0.1179 (2)	0.0422 (4)	0.0078 (3)	0.0610 (10)	
O3	0.0358 (3)	0.3316 (5)	0.1821 (3)	0.0735 (13)	
O4	−0.0384 (3)	0.2223 (5)	0.2512 (3)	0.0693 (12)	
C1	0.1739 (3)	−0.0072 (6)	0.0016 (4)	0.0560 (13)	
C2	0.2274 (4)	0.0195 (8)	0.0668 (5)	0.084 (2)	
H2A	0.2129	−0.0175	0.1172	0.1*	
H2B	0.2279	0.1032	0.076	0.1*	
C3	0.0926 (6)	0.0203 (9)	−0.1995 (7)	0.102 (3)	
H3A	0.0675	0.0052	−0.2497	0.123*	
H3B	0.0622	0.064	−0.1636	0.123*	
C4	0.1574 (7)	0.0968 (12)	−0.2194 (9)	0.104 (4)	
H4B	0.1797	0.1187	−0.1686	0.149*	
H4A	0.19	0.0501	−0.2504	0.149*	
C5	0.1422 (12)	0.1986 (18)	−0.2636 (13)	0.110 (10)	
H5A	0.1529	0.1863	−0.3202	0.301*	
H5B	0.1694	0.2623	−0.243	0.301*	
H5C	0.094	0.2167	−0.258	0.301*	
C6	0.1961 (3)	−0.2438 (6)	−0.1993 (4)	0.0638 (14)	
C7	0.2427 (5)	−0.3094 (6)	−0.1541 (8)	0.085 (2)	
H7	0.2404	−0.3074	−0.0975	0.102*	
C8	0.2919 (5)	−0.3771 (8)	−0.1913 (7)	0.097 (3)	
H8	0.3219	−0.4216	−0.1597	0.117*	
C9	0.2973 (6)	−0.3797 (9)	−0.2740 (8)	0.109 (4)	
H9	0.3301	−0.4271	−0.2991	0.131*	
C10	0.2531 (7)	−0.3105 (12)	−0.3208 (7)	0.119 (4)	
H10	0.2583	−0.3079	−0.3772	0.143*	
C11	0.2017 (5)	−0.2460 (9)	−0.2835 (5)	0.089 (2)	
H11	0.1706	−0.2036	−0.3152	0.106*	
C12	0.0543 (4)	−0.2221 (7)	−0.0539 (4)	0.0657 (16)	
C13	0.0797 (4)	−0.3076 (9)	−0.0027 (6)	0.089 (3)	
H13	0.1259	−0.3297	−0.0065	0.107*	
C14	0.0377 (6)	−0.3603 (10)	0.0536 (6)	0.104 (3)	
H14	0.0558	−0.4189	0.0867	0.125*	
C15	−0.0302 (7)	−0.3290 (12)	0.0625 (8)	0.115 (4)	
H15	−0.0578	−0.3652	0.1015	0.138*	
C16	−0.0567 (5)	−0.2440 (12)	0.0135 (9)	0.113 (3)	
H16	−0.1027	−0.2215	0.0196	0.136*	
C17	−0.0146 (4)	−0.1885 (8)	−0.0475 (6)	0.086 (2)	
H17	−0.033	−0.1317	−0.0819	0.103*	
C18	−0.0086 (4)	0.3153 (7)	0.2375 (5)	0.0694 (17)	
C19	−0.0275 (7)	0.4147 (9)	0.2922 (7)	0.115 (4)	
H19A	−0.0141	0.3942	0.3474	0.138*	
H19B	−0.0774	0.4229	0.2916	0.138*	
C20	−0.0249 (4)	0.1638 (9)	0.0359 (7)	0.111 (2)	
H20A	−0.0138	0.1239	−0.0146	0.109*	
H20B	−0.0535	0.1114	0.0679	0.109*	
C21	−0.0660 (8)	0.2664 (17)	0.0160 (15)	0.100 (10)	
H21B	−0.1011	0.2378	−0.0213	0.24*	
H21A	−0.0902	0.2852	0.0661	0.24*	
C22	−0.0485 (13)	0.3780 (15)	−0.0125 (16)	0.114 (12)	
H22A	−0.0898	0.4246	−0.0170	0.269*	
H22B	−0.0184	0.4163	0.0262	0.269*	
H22C	−0.0252	0.3725	−0.0641	0.269*	
C23	0.1495 (4)	0.2993 (6)	0.0543 (5)	0.0678 (16)	
C24	0.1565 (7)	0.3199 (12)	−0.0289 (6)	0.114 (4)	
H24	0.1239	0.29	−0.0651	0.137*	
C25	0.2113 (8)	0.3843 (15)	−0.0583 (9)	0.113 (6)	
H25	0.2139	0.4012	−0.1137	0.183*	
C26	0.2619 (6)	0.4234 (12)	−0.0071 (9)	0.110 (4)	
H26	0.3002	0.4624	−0.0279	0.156*	
C27	0.2559 (5)	0.4052 (9)	0.0734 (7)	0.111 (4)	
H27	0.29	0.433	0.1085	0.133*	
C28	0.1991 (4)	0.3451 (6)	0.1053 (7)	0.087 (2)	
H28	0.1948	0.336	0.1615	0.105*	
C29	0.0973 (4)	0.0755 (8)	0.1974 (5)	0.0716 (19)	
C30	0.0815 (6)	−0.0416 (9)	0.1932 (7)	0.107 (4)	
H30	0.0583	−0.0716	0.1482	0.128*	
C31	0.1009 (9)	−0.1152 (15)	0.2576 (11)	0.117 (8)	
H31	0.0892	−0.194	0.2555	0.188*	
C32	0.1358 (9)	−0.0744 (19)	0.3219 (9)	0.107 (8)	
H32	0.1488	−0.1251	0.3635	0.188*	
C33	0.1525 (7)	0.0414 (16)	0.3269 (6)	0.113 (5)	
H33	0.1775	0.0691	0.3714	0.159*	
C34	0.1322 (5)	0.1175 (11)	0.2659 (5)	0.099 (3)	
H34	0.1418	0.1969	0.2705	0.119*	

### Atomic displacement parameters (Å^2^)

**Table:**

	*U*^11^	*U*^22^	*U*^33^	*U*^12^	*U*^13^	*U*^23^
Sn1	0.05910 (19)	0.0703 (2)	0.05297 (19)	−0.00035 (18)	−0.0032 (2)	−0.0007 (2)
Sn2	0.05046 (16)	0.0652 (2)	0.05397 (19)	−0.00106 (16)	0.00380 (19)	0.0087 (2)
Cl1	0.0545 (8)	0.165 (2)	0.0727 (11)	0.0156 (11)	−0.0042 (8)	−0.0015 (12)
Cl2	0.304 (6)	0.099 (2)	0.140 (3)	−0.048 (3)	0.074 (4)	−0.015 (2)
O1	0.061 (2)	0.085 (3)	0.060 (2)	0.005 (2)	−0.007 (2)	−0.018 (2)
O2	0.051 (2)	0.070 (3)	0.062 (2)	0.0060 (19)	−0.0063 (19)	−0.011 (2)
O3	0.078 (3)	0.074 (3)	0.068 (3)	−0.006 (2)	0.022 (3)	0.000 (2)
O4	0.064 (3)	0.079 (3)	0.065 (3)	−0.006 (2)	0.018 (2)	−0.004 (2)
C1	0.049 (3)	0.065 (3)	0.054 (3)	−0.002 (2)	0.000 (2)	−0.004 (3)
C2	0.058 (3)	0.111 (6)	0.082 (4)	0.017 (4)	−0.017 (3)	−0.032 (4)
C3	0.115 (7)	0.094 (6)	0.098 (6)	−0.014 (5)	−0.038 (6)	0.024 (5)
C4	0.121 (9)	0.122 (9)	0.129 (10)	0.007 (7)	−0.020 (7)	0.025 (8)
C5	0.12 (2)	0.169 (16)	0.193 (19)	0.012 (15)	0.064 (18)	0.072 (15)
C6	0.060 (3)	0.068 (4)	0.063 (3)	−0.004 (3)	−0.003 (3)	−0.007 (3)
C7	0.087 (5)	0.088 (4)	0.081 (5)	0.009 (4)	0.009 (5)	0.009 (5)
C8	0.083 (5)	0.087 (6)	0.122 (8)	0.008 (4)	0.023 (5)	0.003 (5)
C9	0.095 (6)	0.096 (7)	0.137 (10)	−0.007 (5)	0.031 (7)	−0.038 (7)
C10	0.114 (8)	0.156 (11)	0.087 (7)	0.003 (8)	0.014 (6)	−0.046 (7)
C11	0.097 (6)	0.112 (7)	0.058 (4)	0.001 (5)	0.012 (4)	−0.013 (4)
C12	0.061 (3)	0.083 (4)	0.053 (3)	−0.005 (3)	−0.002 (3)	−0.003 (3)
C13	0.071 (4)	0.125 (8)	0.072 (5)	0.006 (5)	0.001 (4)	0.022 (5)
C14	0.116 (8)	0.110 (7)	0.086 (6)	−0.009 (6)	−0.003 (6)	0.031 (5)
C15	0.105 (8)	0.143 (10)	0.098 (7)	−0.012 (7)	0.031 (6)	0.014 (7)
C16	0.078 (5)	0.120 (8)	0.141 (10)	−0.002 (6)	0.034 (6)	0.002 (8)
C17	0.069 (4)	0.094 (5)	0.095 (6)	0.009 (4)	0.005 (4)	0.008 (5)
C18	0.072 (4)	0.076 (4)	0.061 (4)	0.006 (3)	0.012 (3)	−0.003 (3)
C19	0.110 (10)	0.080 (5)	0.104 (7)	0.004 (6)	0.058 (7)	−0.008 (5)
C20	0.092 (4)	0.108 (6)	0.102 (6)	0.000 (4)	−0.022 (4)	−0.004 (5)
C21	0.105 (12)	0.119 (14)	0.20 (3)	0.001 (10)	−0.0207 (14)	0.090 (17)
C22	0.12 (2)	0.117 (12)	0.30 (3)	−0.018 (13)	−0.09 (2)	0.089 (16)
C23	0.061 (3)	0.069 (4)	0.074 (4)	−0.009 (3)	0.017 (3)	0.008 (3)
C24	0.113 (7)	0.160 (11)	0.070 (5)	−0.055 (7)	0.017 (5)	0.004 (6)
C25	0.160 (12)	0.180 (13)	0.117 (10)	−0.073 (11)	0.054 (9)	0.021 (9)
C26	0.103 (7)	0.125 (9)	0.163 (12)	−0.044 (7)	0.047 (8)	0.009 (8)
C27	0.093 (6)	0.103 (6)	0.137 (10)	−0.046 (5)	0.005 (6)	0.006 (6)
C28	0.090 (5)	0.080 (4)	0.092 (5)	−0.026 (3)	−0.009 (5)	0.009 (5)
C29	0.060 (3)	0.091 (5)	0.064 (4)	0.019 (3)	0.015 (3)	0.017 (4)
C30	0.125 (8)	0.093 (6)	0.102 (7)	0.047 (6)	0.046 (6)	0.028 (5)
C31	0.120 (14)	0.143 (12)	0.148 (13)	0.086 (11)	0.079 (12)	0.083 (11)
C32	0.166 (13)	0.206 (17)	0.099 (9)	0.105 (13)	0.055 (9)	0.074 (10)
C33	0.111 (8)	0.215 (16)	0.072 (6)	0.054 (10)	0.012 (5)	0.033 (8)
C34	0.090 (5)	0.147 (9)	0.060 (5)	0.026 (6)	0.010 (4)	0.020 (5)

### Geometric parameters (Å, °)

**Table:**

Sn1—C3	2.105 (9)	C13—H13	0.93
Sn1—C12	2.125 (7)	C14—C15	1.368 (17)
Sn1—C6	2.137 (7)	C14—H14	0.93
Sn1—O1	2.178 (4)	C15—C16	1.362 (17)
Sn1—O4^i^	2.478 (5)	C15—H15	0.93
Sn2—C29	2.129 (7)	C16—C17	1.437 (14)
Sn2—C23	2.130 (6)	C16—H16	0.93
Sn2—C20	2.130 (8)	C17—H17	0.93
Sn2—O3	2.190 (5)	C18—C19	1.493 (12)
Sn2—O2	2.469 (5)	C19—H19A	0.97
Cl1—C2	1.746 (7)	C19—H19B	0.97
Cl2—C19	1.699 (11)	C20—C21	1.455 (17)
O1—C1	1.257 (8)	C20—H20A	0.97
O2—C1	1.224 (7)	C20—H20B	0.97
O3—C18	1.264 (9)	C21—C22	1.40 (3)
O4—C18	1.231 (9)	C21—H21B	0.97
O4—Sn1^ii^	2.478 (5)	C21—H21A	0.97
C1—C2	1.518 (9)	C22—H22A	0.96
C2—H2A	0.97	C22—H22B	0.96
C2—H2B	0.97	C22—H22C	0.96
C3—C4	1.562 (16)	C23—C28	1.376 (12)
C3—H3A	0.97	C23—C24	1.392 (12)
C3—H3B	0.97	C24—C25	1.376 (15)
C4—C5	1.40 (2)	C24—H24	0.93
C4—H4B	0.97	C25—C26	1.36 (2)
C4—H4A	0.97	C25—H25	0.93
C5—H5A	0.96	C26—C27	1.342 (16)
C5—H5B	0.96	C26—H26	0.93
C5—H5C	0.96	C27—C28	1.396 (12)
C6—C11	1.384 (10)	C27—H27	0.93
C6—C7	1.387 (12)	C28—H28	0.93
C7—C8	1.370 (13)	C29—C30	1.375 (14)
C7—H7	0.93	C29—C34	1.394 (14)
C8—C9	1.360 (17)	C30—C31	1.401 (16)
C8—H8	0.93	C30—H30	0.93
C9—C10	1.394 (18)	C31—C32	1.34 (3)
C9—H9	0.93	C31—H31	0.93
C10—C11	1.381 (15)	C32—C33	1.37 (2)
C10—H10	0.93	C32—H32	0.93
C11—H11	0.93	C33—C34	1.383 (15)
C12—C13	1.378 (12)	C33—H33	0.93
C12—C17	1.391 (11)	C34—H34	0.93
C13—C14	1.370 (14)		
			
C3—Sn1—C12	123.9 (4)	C15—C14—C13	121.6 (10)
C3—Sn1—C6	117.4 (4)	C15—C14—H14	119.2
C12—Sn1—C6	117.3 (3)	C13—C14—H14	119.2
C3—Sn1—O1	98.6 (3)	C16—C15—C14	119.1 (10)
C12—Sn1—O1	93.0 (2)	C16—C15—H15	120.5
C6—Sn1—O1	90.1 (2)	C14—C15—H15	120.5
C3—Sn1—O4^i^	83.8 (3)	C15—C16—C17	120.9 (10)
C12—Sn1—O4^i^	88.0 (2)	C15—C16—H16	119.6
C6—Sn1—O4^i^	86.2 (2)	C17—C16—H16	119.6
O1—Sn1—O4^i^	176.21 (18)	C12—C17—C16	118.2 (9)
C29—Sn2—C23	116.2 (3)	C12—C17—H17	120.9
C29—Sn2—C20	120.0 (4)	C16—C17—H17	120.9
C23—Sn2—C20	121.8 (4)	O4—C18—O3	125.3 (7)
C29—Sn2—O3	94.5 (3)	O4—C18—C19	115.7 (7)
C23—Sn2—O3	90.1 (2)	O3—C18—C19	119.0 (8)
C20—Sn2—O3	99.1 (3)	C18—C19—Cl2	116.7 (7)
C29—Sn2—O2	86.4 (2)	C18—C19—H19A	108.1
C23—Sn2—O2	84.4 (2)	Cl2—C19—H19A	108.1
C20—Sn2—O2	85.3 (3)	C18—C19—H19B	108.1
O3—Sn2—O2	174.26 (18)	Cl2—C19—H19B	108.1
C1—O1—Sn1	121.9 (4)	H19A—C19—H19B	107.3
C1—O2—Sn2	134.8 (4)	C21—C20—Sn2	117.6 (9)
C18—O3—Sn2	121.9 (5)	C21—C20—H20A	107.7
C18—O4—Sn1^ii^	137.8 (5)	Sn2—C20—H20A	107.9
O2—C1—O1	125.9 (6)	C21—C20—H20B	108
O2—C1—C2	116.8 (6)	Sn2—C20—H20B	107.9
O1—C1—C2	117.3 (5)	H20A—C20—H20B	107.2
C1—C2—Cl1	115.5 (5)	C22—C21—C20	132.7 (16)
C1—C2—H2A	108.4	C22—C21—H21B	104.8
Cl1—C2—H2A	108.4	C20—C21—H21B	104.8
C1—C2—H2B	108.4	C22—C21—H21A	101.0
Cl1—C2—H2B	108.4	C20—C21—H21A	104.5
H2A—C2—H2B	107.5	H21B—C21—H21A	105.7
C4—C3—Sn1	113.6 (8)	C21—C22—H22A	109.2
C4—C3—H3A	108.8	C21—C22—H22B	109.6
Sn1—C3—H3A	108.9	H22A—C22—H22B	107.0
C4—C3—H3B	108.9	C21—C22—H22C	109.6
Sn1—C3—H3B	108.9	H22A—C22—H22C	111.0
H3A—C3—H3B	107.7	H22B—C22—H22C	109.5
C5—C4—C3	113.9 (13)	C28—C23—C24	117.6 (8)
C5—C4—H4B	108.8	C28—C23—Sn2	120.9 (6)
C3—C4—H4B	108.8	C24—C23—Sn2	121.4 (6)
C5—C4—H4A	108.8	C25—C24—C23	120.5 (11)
C3—C4—H4A	108.8	C25—C24—H24	119.7
H4B—C4—H4A	107.7	C23—C24—H24	119.7
C4—C5—H5A	109.5	C26—C25—C24	120.8 (12)
C4—C5—H5B	109.4	C26—C25—H25	119.6
H5A—C5—H5B	109.5	C24—C25—H25	119.6
C4—C5—H5C	109.5	C27—C26—C25	119.5 (9)
H5A—C5—H5C	109.5	C27—C26—H26	120.3
H5B—C5—H5C	109.5	C25—C26—H26	120.2
C11—C6—C7	118.1 (8)	C26—C27—C28	120.8 (11)
C11—C6—Sn1	119.4 (6)	C26—C27—H27	119.6
C7—C6—Sn1	122.5 (6)	C28—C27—H27	119.6
C8—C7—C6	121.3 (11)	C23—C28—C27	120.6 (10)
C8—C7—H7	119.4	C23—C28—H28	119.7
C6—C7—H7	119.4	C27—C28—H28	119.7
C9—C8—C7	120.6 (11)	C30—C29—C34	118.9 (9)
C9—C8—H8	119.7	C30—C29—Sn2	120.6 (7)
C7—C8—H8	119.7	C34—C29—Sn2	120.5 (7)
C8—C9—C10	119.2 (10)	C29—C30—C31	119.2 (15)
C8—C9—H9	120.4	C29—C30—H30	120.4
C10—C9—H9	120.4	C31—C30—H30	120.4
C11—C10—C9	120.1 (10)	C32—C31—C30	121.3 (17)
C11—C10—H10	119.9	C32—C31—H31	119.3
C9—C10—H10	120	C30—C31—H31	119.4
C10—C11—C6	120.5 (10)	C31—C32—C33	120.4 (12)
C10—C11—H11	119.8	C31—C32—H32	119.9
C6—C11—H11	119.7	C33—C32—H32	119.8
C13—C12—C17	119.5 (8)	C32—C33—C34	120.0 (14)
C13—C12—Sn1	122.1 (6)	C32—C33—H33	120
C17—C12—Sn1	118.4 (6)	C34—C33—H33	120
C14—C13—C12	120.8 (9)	C33—C34—C29	120.1 (13)
C14—C13—H13	119.6	C33—C34—H34	120
C12—C13—H13	119.6	C29—C34—H34	119.9
			
C3—Sn1—O1—C1	63.3 (6)	C14—C15—C16—C17	1(2)
C12—Sn1—O1—C1	−61.6 (6)	C13—C12—C17—C16	0.8 (14)
C6—Sn1—O1—C1	−178.9 (5)	Sn1—C12—C17—C16	−179.9 (8)
C29—Sn2—O2—C1	−55.1 (6)	C15—C16—C17—C12	−1.5 (18)
C23—Sn2—O2—C1	61.7 (6)	Sn1^ii^—O4—C18—O3	172.1 (5)
C20—Sn2—O2—C1	−175.6 (7)	Sn1^ii^—O4—C18—C19	−7.7 (13)
C29—Sn2—O3—C18	−54.2 (6)	Sn2—O3—C18—O4	−1.5 (11)
C23—Sn2—O3—C18	−170.5 (6)	Sn2—O3—C18—C19	178.3 (8)
C20—Sn2—O3—C18	67.2 (7)	O4—C18—C19—Cl2	−175.0 (8)
Sn2—O2—C1—O1	−175.3 (5)	O3—C18—C19—Cl2	5.2 (15)
Sn2—O2—C1—C2	4.0 (10)	C29—Sn2—C20—C21	138.4 (13)
Sn1—O1—C1—O2	−8.1 (10)	C23—Sn2—C20—C21	−58.1 (14)
Sn1—O1—C1—C2	172.6 (5)	O3—Sn2—C20—C21	37.7 (14)
O2—C1—C2—Cl1	−166.5 (6)	O2—Sn2—C20—C21	−138.5 (14)
O1—C1—C2—Cl1	12.8 (10)	Sn2—C20—C21—C22	45 (4)
C12—Sn1—C3—C4	145.3 (8)	C29—Sn2—C23—C28	−36.4 (7)
C6—Sn1—C3—C4	−48.8 (10)	C20—Sn2—C23—C28	159.5 (6)
O1—Sn1—C3—C4	45.7 (9)	O3—Sn2—C23—C28	58.7 (7)
O4^i^—Sn1—C3—C4	−131.3 (9)	O2—Sn2—C23—C28	−119.6 (7)
Sn1—C3—C4—C5	174.0 (13)	C29—Sn2—C23—C24	139.1 (9)
C3—Sn1—C6—C11	−36.3 (8)	C20—Sn2—C23—C24	−25.0 (10)
C12—Sn1—C6—C11	130.5 (7)	O3—Sn2—C23—C24	−125.7 (9)
O1—Sn1—C6—C11	−136.0 (7)	O2—Sn2—C23—C24	55.9 (9)
O4^i^—Sn1—C6—C11	44.7 (7)	C28—C23—C24—C25	0(2)
C3—Sn1—C6—C7	143.5 (6)	Sn2—C23—C24—C25	−175.8 (12)
C12—Sn1—C6—C7	−49.6 (7)	C23—C24—C25—C26	4(3)
O1—Sn1—C6—C7	43.9 (6)	C24—C25—C26—C27	−4(3)
O4^i^—Sn1—C6—C7	−135.4 (6)	C25—C26—C27—C28	1(2)
C11—C6—C7—C8	−1.6 (12)	C24—C23—C28—C27	−3.0 (14)
Sn1—C6—C7—C8	178.5 (7)	Sn2—C23—C28—C27	172.7 (8)
C6—C7—C8—C9	1.4 (14)	C26—C27—C28—C23	2.6 (17)
C7—C8—C9—C10	1.5 (17)	C23—Sn2—C29—C30	−129.2 (6)
C8—C9—C10—C11	−4.1 (18)	C20—Sn2—C29—C30	35.2 (7)
C9—C10—C11—C6	3.8 (18)	O3—Sn2—C29—C30	138.5 (6)
C7—C6—C11—C10	−1.0 (14)	O2—Sn2—C29—C30	−47.2 (6)
Sn1—C6—C11—C10	178.9 (8)	C23—Sn2—C29—C34	50.7 (7)
C3—Sn1—C12—C13	−161.3 (7)	C20—Sn2—C29—C34	−144.9 (7)
C6—Sn1—C12—C13	32.7 (8)	O3—Sn2—C29—C34	−41.7 (6)
O1—Sn1—C12—C13	−58.9 (7)	O2—Sn2—C29—C34	132.6 (6)
O4^i^—Sn1—C12—C13	117.4 (7)	C34—C29—C30—C31	0.0 (13)
C3—Sn1—C12—C17	19.4 (8)	Sn2—C29—C30—C31	179.8 (8)
C6—Sn1—C12—C17	−146.6 (6)	C29—C30—C31—C32	−1.7 (18)
O1—Sn1—C12—C17	121.8 (7)	C30—C31—C32—C33	1(2)
O4^i^—Sn1—C12—C17	−61.9 (7)	C31—C32—C33—C34	1(2)
C17—C12—C13—C14	0.6 (15)	C32—C33—C34—C29	−2.8 (16)
Sn1—C12—C13—C14	−178.7 (8)	C30—C29—C34—C33	2.3 (13)
C12—C13—C14—C15	−1.3 (18)	Sn2—C29—C34—C33	−177.6 (7)
C13—C14—C15—C16	1(2)		

Symmetry codes: (i) −*x*, −*y*, *z*−1/2; (ii) −*x*, −*y*, *z*+1/2.
